# AI-driven virtual standardized patients combined with scenario-based simulation in anesthesiology training: a randomized pilot study

**DOI:** 10.3389/fmed.2026.1927674

**Published:** 2026-09-08

**Authors:** Xiaohua Wang, Aming Sang, Jing Zhang, Hexiao Tang, Yujia Liu, Zirui Zhao, Jinping Liu, Ming Xu, Xinyi Li

**Affiliations:** 1 Department of Anesthesiology, Zhongnan Hospital of Wuhan University, Wuhan, China; 2 Department of Thoracic Surgery, Zhongnan Hospital of Wuhan University, Wuhan, China; 3 School of Basic Medical Sciences, Wuhan University, Wuhan, Hubei Province, China; 4 Department of Cardiovascular Surgery, Zhongnan Hospital of Wuhan University, Wuhan, China

**Keywords:** anesthesiology education, artificial intelligence, clinical simulation, crisis resource management, non-technical skills, short-term simulated performance, virtual standardized patient

## Abstract

**Background:**

Anesthesiology trainees require repeated opportunities to practice perioperative crisis recognition, decision-making, communication and teamwork, which are central to crisis resource management (CRM) and non-technical skills. High-fidelity simulation supports these competencies but is resource intensive to deliver repeatedly. AI-driven virtual standardized patients (AI-VSPs) may complement simulation and structured debriefing by enabling interactive practice and feedback.

**Methods:**

We conducted a prospective, randomized, three-arm pilot study among 60 students enrolled in a professional master's degree program in anesthesiology at Zhongnan Hospital of Wuhan University between January and June 2026. Participants were assigned to conventional teaching (group A, *n* = 20), scenario-based simulation with structured debriefing (group B, *n* = 20), or the same simulation pathway with AI-VSP training (group C, *n* = 20); each group received 16 h of teaching. In group C, four 1-hour AI-VSP sessions replaced 4 h of conventional case discussion. The primary outcome was short-term simulated clinical performance measured using a locally developed 100-point competency rubric. Secondary outcomes were theoretical examination, skills examination and teaching satisfaction, with satisfaction interpreted as acceptability rather than effectiveness.

**Results:**

All participants completed the study and were analyzed. No statistically significant between-group differences were detected in the reported baseline characteristics. In participant-level post-intervention analyses, total simulated clinical performance was higher in group C than in groups A and B (88.2 ± 4.8 vs. 78.0 ± 7.7 and 80.4 ± 6.5; F(2, 57) = 13.72, *P* < 0.001, partial *η*^2^ = 0.325). Bonferroni-adjusted comparisons favored group C for clinical decision-making, communication, total performance, theoretical examination, skills examination and teaching satisfaction.

**Conclusions:**

A time-matched pathway in which AI-VSP practice replaced part of conventional case discussion while simulation and structured debriefing were retained was associated with better short-term performance on aligned anesthesiology crisis tasks. These findings support the feasibility of this approach and suggest potential benefits in selected CRM-relevant domains, but do not establish comprehensive CRM competence, definitive effectiveness or durable transfer to clinical practice. Larger multicenter studies with prespecified analyses, detailed reporting of AI-system characteristics, delayed assessment and workplace-based outcomes are needed.

## Introduction

Anesthesiology is central to perioperative medicine, where clinicians must respond to rapidly changing physiology, high-acuity events and evolving team demands. Safe care depends on early crisis recognition, timely decision-making, closed-loop communication and coordinated team action (1, 2). These capabilities are central to crisis resource management (CRM), an established framework emphasizing situation awareness, decision-making, task management, leadership, teamwork and communication during high-acuity events (3, 4). In China, clinical medicine professional master's degree students undertake a three-year postgraduate program integrated with standardized residency training in accordance with national guidance (5, 6). This integrated pathway requires trainees to develop clinical reasoning and crisis-management skills while completing postgraduate coursework and supervised clinical rotations.

Traditional anesthesiology teaching still relies on lectures, bedside instruction, supervised clinical work and case discussion (7–9). These methods remain essential for foundational knowledge and professional socialization, but they provide limited exposure to low-frequency, high-risk perioperative emergencies. Real crises are unpredictable and cannot be repeatedly reproduced for teaching, and patient-safety and ethical constraints limit opportunities for novice trainees to practice crisis management in real time. Accordingly, an important educational gap is how to provide repeated, feedback-rich practice in CRM-relevant cognitive and communication processes when opportunities for repeated high-fidelity simulation are limited.

Scenario-based simulation addresses part of this gap by creating controlled, repeatable and psychologically safe environments for practicing technical and non-technical skills. Within anesthesiology, these activities map closely onto CRM learning, particularly situation assessment, decision-making, task management, teamwork and communication (3, 4, 10). Simulation-based medical education emphasizes repetitive practice, feedback, mastery learning and deliberate practice as mechanisms for skill acquisition (8, 11–16). Structured debriefing, including the PEARLS approach, supports reflective learning by helping trainees identify cognitive and behavioral gaps after simulated events (17). Hybrid simulation approaches may further strengthen self-efficacy and engagement when structured scenario exposure is combined with interactive preparation (18).

Virtual patients and VSPs are increasingly used in health professions education to support clinical reasoning, history taking and repeated case-based practice (19, 20). Large language models and other AI-enabled systems have expanded the potential for interactive virtual patient dialogue and automated formative feedback (21–25). These opportunities also bring important implementation risks: AI-enabled educational interventions require transparent reporting, careful supervision, privacy safeguards and explicit controls for hallucination, automation bias and learner over-trust (23, 24, 26–28).

Within this framework, we conceptualized AI-driven VSP training as an embedded component of the combined scenario-based simulation and structured-debriefing pathway rather than as a separate add-on. The trial therefore used a time-matched, nested component-comparison design to examine the educational signals associated with substituting scenario-based simulation and structured debriefing for part of conventional teaching (group B vs. group A), and with substituting four 1-hour AI-VSP sessions for part of conventional case discussion within the combined pathway (group C vs. group B). Without increasing total teaching time, the AI-VSP component provided repeated interactive practice in selected CRM-relevant cognitive and communication processes.

This pilot study evaluated an AI-driven VSP combined with scenario-based simulation for anesthesiology professional master's degree students. The objective was to examine whether this integrated model was associated with better short-term performance on aligned crisis tasks containing selected CRM-relevant domains, including clinical decision-making, teamwork and communication. We focused on short-term simulated clinical performance in aligned perioperative bradycardia and hypotension crisis tasks, rather than broad clinical competency transfer. The study compared conventional teaching, scenario-based simulation with structured debriefing, and scenario-based simulation combined with AI-driven VSP training.

## Materials and methods

### Study design, setting, and reporting framework

We conducted a prospective randomized parallel-group pilot study in the Department of Anesthesiology, Zhongnan Hospital of Wuhan University, from January 2026 to June 2026. Reporting followed CONSORT 2025 principles for randomized trials, TIDieR principles for intervention description, and CONSORT-AI/SPIRIT-AI principles for interventions with an AI component (26, 27, 29–31). Because this was an institutional educational pilot study, the emphasis was on feasibility, short-term simulated performance and preliminary effect estimation, not confirmatory effectiveness. The study was not prospectively registered.

The study was approved by the Ethics Committee of Zhongnan Hospital of Wuhan University before participant enrollment (Approval No. 2026170K). All participants provided written informed consent before enrollment.

### Participants and sample size

At our institution, anesthesiology professional master's degree students are postgraduate medical trainees enrolled in a three-year, full-time, clinically oriented program integrated with China's standardized residency training. Following undergraduate medical education, trainees undertake supervised clinical rotations while completing postgraduate coursework and research or thesis requirements. Participants in the present study were enrolled at the beginning of their first specialty-specific rotation in the Department of Anesthesiology and had not yet completed the structured anesthesiology practice modules.

Inclusion criteria were: (1) completion of prerequisite anesthesiology coursework with passing grades; (2) a score of at least 60/100 on the pre-rotation theoretical examination, which operationally defined basic clinical cognitive ability; (3) no previous completion of a formal anesthesiology specialty rotation or structured department-based module in operating-room anesthesia, procedural skills, or perioperative crisis management. Eligibility was confirmed by the teaching coordinator using academic and rotation records. Exclusion criteria were withdrawal before completion of training or missing key assessment data.

The target sample was 60 participants, with 20 per arm, based on the number of eligible students rotating through the department during the study period and the exploratory nature of the intervention. No formal *a priori* power calculation was performed. The study was designed to estimate preliminary effects, identify reporting and implementation issues, and inform a future adequately powered multicenter trial.

### Randomization, allocation concealment, and masking

Sixty eligible students were assigned in a 1:1:1 ratio to group A, group B or group C using a random-number table prepared by a researcher who was not involved in teaching delivery or outcome assessment. Before enrollment began, group assignments were placed in sequentially numbered, opaque, sealed envelopes. A teaching coordinator enrolled participants and opened the next envelope only after consent and baseline data collection were complete. The allocation sequence was not accessible to instructors or outcome raters before assignment.

Instructors and learners could not be blinded because of the nature of the educational intervention. Outcome ratings were performed from coded video recordings by two associate chief anesthesiologists who were not involved in intervention delivery. Video files were stripped of group identifiers, coded by a research assistant, and presented to raters in a randomized mixed order, so raters were blinded to group allocation and assessment time point.

### Teaching interventions

To minimize confounding by teaching exposure, total instructional time was fixed at 16 h in all three groups. The study used a time-matched, nested component-comparison design in which selected teaching activities were substituted rather than added. All groups addressed the same learning objectives: recognition of perioperative bradycardia and hypotension, systematic initial assessment, interpretation of physiological and monitoring information, identification of likely causes, prioritization of initial management, communication of clinical decisions, and reassessment of treatment response.

Departmental orientation and workflow training comprised operating-room safety procedures, patient identification and handover, preoperative assessment and documentation, anesthesia workstation and monitor checks, medication-safety procedures, and the local emergency-escalation process. Conventional core content referred to this orientation together with thematic instruction and supervised clinical teaching on the recognition and initial management of perioperative bradycardia and hypotension. Instructors were attending anesthesiologists or senior faculty members with at least 5 years of teaching experience.

Simulation setting and participant configuration. The scenario-based training and the pre- and post-intervention assessments were conducted in a simulated operating room at the Clinical Skills Training Center of Zhongnan Hospital of Wuhan University. Each learner participated individually in the role of the primary anesthesiology trainee. The nurse and surgeon were predefined simulated roles generated within the scenario environment rather than learner teammates, standardized patients, human actors, or confederates. Across participants, the same case scripts, role prompts, trigger points, vital-sign trajectories, monitor alarms, cue sequences, available clinical information, and stopping criteria were used. Faculty supervised scenario delivery but did not perform an in-scenario team role.

Group A: conventional teaching. Group A received the conventional core content together with faculty-guided case discussion. The common learning objectives were addressed through thematic instruction, supervised clinical teaching, and analysis of written clinical cases, without high-fidelity simulation or AI-driven VSP training.

Group B: scenario-based simulation with structured debriefing. Within the same 16-hour total teaching exposure as group A, group B retained the conventional core content, while high-fidelity scenario-based simulation and structured GAS debriefing replaced an equivalent portion of conventional case-discussion time. The simulation module focused on recognition and management of perioperative bradycardia and hypotension crisis. Each scenario was followed by structured GAS debriefing: gathering learner perceptions and events, analyzing clinical reasoning and team communication, and summarizing take-home points and improvement plans. Facilitators used a standardized debriefing checklist to support debriefing fidelity. The simulation and GAS debriefing mapped onto CRM-relevant processes, including situation assessment, treatment prioritization, task management, closed-loop communication, and team coordination.

**Figure 1:**
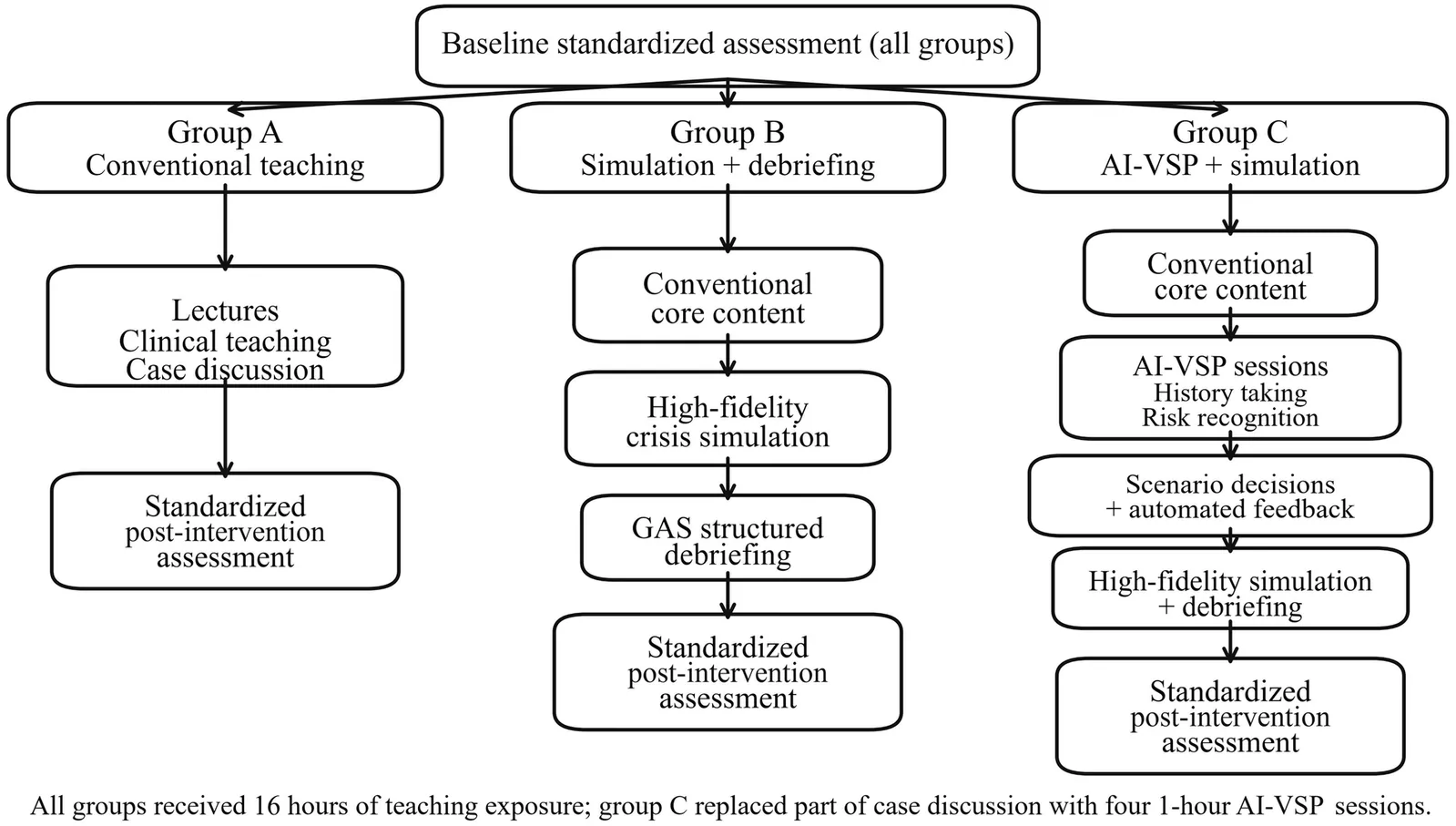
Structure of the three teaching interventions. A common baseline standardized assessment preceded allocation-specific teaching, and all groups completed a standardized post-intervention assessment. All groups received 16 h of teaching. Group B substituted high-fidelity scenario-based simulation and structured debriefing for an equivalent portion of conventional case-discussion time. Group C retained the simulation and debriefing components of group B but replaced 4 h of conventional case discussion with four 1-hour AI-driven VSP sessions. The intervention arms therefore represented time-matched component substitutions rather than additive teaching exposure. AI, artificial intelligence; GAS, Gather, Analyze, Summarize; VSP, virtual standardized patient.

Group C: scenario-based simulation with AI-driven VSP training. Group C retained the conventional core content, high-fidelity simulation, and structured GAS debriefing used in group B, including the same three-phase format, checklist, and faculty facilitation procedures. Within the same 16-hour teaching exposure, four 1-hour AI-VSP sessions using CliniTrek replaced 4 h of conventional case discussion. Each session was completed individually; learners interacted by voice with a virtual patient and case-specific software agents rather than learner teammates or human actors. The sessions addressed the same learning objectives as the simulation, with emphasis on preoperative information gathering, risk recognition, interpretation of perioperative vital signs, and decision-making under time pressure. After each session, faculty reviewed the automated report with the learner; this formative feedback was separate from and did not alter the GAS debriefing after high-fidelity simulation. The intervention framework is shown in Figure 1.

### AI-VSP system and safeguards

The AI-VSP platform used in this study was CliniTrek, a generative AI-driven clinician–patient intelligent interaction and competency assessment system developed by Wuhan Kelin Siyuan Information Technology Co., Ltd. (Wuhan, China). It was used as a faculty-supervised formative simulation tool rather than as a clinical decision-support system or an autonomous outcome assessor. The platform supported a repeated practice–feedback cycle through three functions: (1) voice-based preoperative interaction, through which learners obtained patient history, comorbidity, and examination information; (2) perioperative decision training involving scripted changes in vital signs and monitor alarms; and (3) checklist-linked automated formative feedback presented in a post-session report.

For this study, CliniTrek was locally deployed on password-protected institutional teaching computers and configured with faculty-authored anesthesiology cases. The locked configuration used from January to June 2026 combined a scenario-constrained large-language-model dialogue engine with a faculty-authored case fact bank, scripted vital-sign trajectories, rule-based trigger events, and feedback templates. Each session was completed individually by one learner. Depending on the case script, the software-mediated roles included a virtual patient, a virtual nurse, and, where relevant, a family agent. The virtual nurse acknowledged management instructions and activated predefined actions and corresponding physiological responses. No learner teammates or human actors participated as in-scenario team members; faculty supervised platform use but did not perform a scenario role. A public model-version identifier was not available, and further technical and governance details are provided in Supplementary Table S4.

Automated feedback was generated from recorded scenario events using predefined rules rather than unrestricted large-language-model judgment. Learner questions and management instructions activated predefined information-release or scenario branches, and recorded actions were mapped to faculty-authored critical-action checklist items and timing and sequence rules. The reports summarized decision timing, omitted critical actions, delayed or potentially unsafe actions, activated error pathways, and template-based improvement prompts. Narrative comments were assembled from faculty-approved feedback templates. The large-language-model component mediated the simulated-patient dialogue but did not independently assign ANTS or CRM scores or determine any study outcome.

The intervention was scenario-constrained and faculty-supervised. Anesthesiology faculty reviewed all case scripts, learning objectives, critical actions and feedback templates before use. Learners were instructed not to enter real patient identifiers. AI responses were limited to the simulated patient role and predefined case facts; vital-sign deterioration and critical trigger events were controlled by scripted scenario rules rather than unrestricted model generation. Faculty reviewed training summaries during debriefing and corrected unsafe, incomplete or off-topic feedback when necessary.

### Training-assessment alignment

All groups were assessed using standardized scenario-based tasks involving perioperative bradycardia and hypotension. The assessment case was parallel but not identical to the training cases: it preserved the same critical action domains, including crisis recognition, airway-breathing-circulation assessment, differential diagnosis, treatment prioritization, team communication and reassessment, but used a different fictional patient profile, comorbidity and distractor information, trigger timing and cue sequence. Therefore, the endpoint is interpreted as short-term performance on aligned simulated crisis tasks rather than broad workplace clinical competence. The assessed domains therefore included both crisis-management actions and selected CRM-relevant non-technical processes.

### Outcome measures

The primary outcome was short-term simulated clinical performance, scored using a 100-point competency rubric developed with reference to the Anaesthetists' Non-Technical Skills system, anesthesiology competency frameworks and established Delphi-method guidance for competency-instrument development (32–35). The rubric covered emergency management, teamwork, clinical decision-making, communication and professionalism, each scored from 0 to 20 points. A two-round Delphi review by five anesthesiology and medical education experts yielded a content validity index of 0.92. The scoring rubric is detailed in Supplementary Table S1. The rubric therefore sampled both crisis-management actions and selected CRM-relevant non-technical domains. Although it was informed by ANTS, it was a locally developed competency rubric rather than the formal ANTS instrument and was not treated as a validated comprehensive measure of CRM competence.

Before the intervention (8–12 January 2026) and after completion of the allocated teaching programs (12–16 June 2026), participants completed standardized parallel scenario-based assessments that were video recorded. Each assessment was completed individually, with the learner acting as the primary anesthesiology trainee under the same role scripts, trigger rules, cue sequence, and physiological-response framework used for all participants. Two blinded raters independently scored the videos after calibration training with sample videos and rubric anchors. The final score was the mean of the two independent ratings. If raters differed by more than 10 points in the total score, they reviewed the predefined rubric anchors and reached consensus for the discrepant domains. Across the 120 pre- and post-intervention videos, inter-rater reliability for total scores was excellent [two-way random-effects, absolute-agreement ICC for the mean of two raters, ICC(A,2) = 0.996; corresponding single-rater ICC(A,1) = 0.992]; no total-score difference exceeded 10 points.

Secondary outcomes were theoretical examination score, skills examination score and teaching satisfaction. The theoretical examination was an individually completed, faculty-developed 100-point written test comprising multiple-choice, short-answer and case-analysis items. It assessed factual and applied knowledge of the recognition and initial management of perioperative bradycardia and hypotension; CRM principles were not assessed as a separate content domain, and behavioural CRM competence was not directly evaluated. The skills examination was an individually completed 100-point structured practical assessment scored with a standardized checklist covering anesthesiology procedures, monitoring interpretation, initial emergency management and reassessment. It assessed individual technical and applied clinical performance and was distinct from the video-recorded primary outcome assessment. Both examinations were administered under faculty supervision using standardized instructions and scoring procedures at the pre- and post-intervention assessment periods described above. Further details are provided in Supplementary Table S5.

Teaching satisfaction was evaluated after the intervention using a locally developed 100-point questionnaire covering teaching content, teaching method, interaction and engagement, perceived learning gain and overall satisfaction. Because the satisfaction questionnaire was self-developed and internal consistency was not calculated, satisfaction is reported only as acceptability/feasibility evidence, not as evidence of educational effectiveness.

### Quality control and intervention fidelity

The same teaching team designed and implemented all course components. Simulation scripts, teaching materials, assessment tasks, scoring standards and debriefing checklists were standardized before study initiation. Data entry was performed independently by two researchers and cross-checked to reduce transcription errors.

No participant withdrew and no key outcome data were missing. All 20 learners assigned to group C completed the four scheduled AI-VSP sessions, corresponding to 4 h of exposure per learner. Attendance logs indicated 100% session completion, and no session was terminated because of technical failure. Session-level completion data and author-confirmed system quality-audit summaries were available. Complete raw transcripts and time-stamped interaction logs were not retained for all learners, which limited independent dialogue-level reanalysis.

### Statistical analysis

Continuous variables are reported as mean ± standard deviation and categorical variables as counts. Baseline continuous variables were compared using one-way analysis of variance. Categorical variables were compared using chi-square or Fisher's exact tests, as appropriate. The primary exploratory analyses compared post-intervention outcomes among groups; therefore, no sphericity assumption was required.

Post-intervention reporting included omnibus F statistics, *P* values, partial eta squared, Bonferroni-adjusted pairwise *P* values, mean differences, 95% confidence intervals and Cohen's *d*. Estimates were calculated from participant-level scores (*n* = 20 per group). Pairwise comparisons used pooled-variance independent-samples t tests with Bonferroni adjustment across the three group contrasts; standardized mean differences used the pooled standard deviation. These analyses are exploratory and hypothesis-generating.

After the primary unadjusted analyses, an exploratory *post hoc* ANCOVA compared post-intervention total clinical performance, theoretical examination and skills examination scores among groups. Each model adjusted for the corresponding baseline score and included group as a fixed effect. Adjusted means and pairwise differences with 95% confidence intervals were reported. Group-by-baseline interaction tests did not indicate departure from homogeneous regression slopes (all *P* ≥ 0.207). This analysis was not prespecified and remains hypothesis-generating. Linear mixed-effects sensitivity analyses were not performed.

Faculty preparation, teaching, debrief/review, equipment, platform and consumable inputs were summarized descriptively in CNY. Pilot cost included all recorded inputs, whereas recurring cost excluded all one-time inputs, including faculty preparation or script-development costs and equipment allocations. These estimates describe implementation burden and are not a formal cost-effectiveness analysis.

## Results

### Participant flow and baseline characteristics

**Figure 2:**
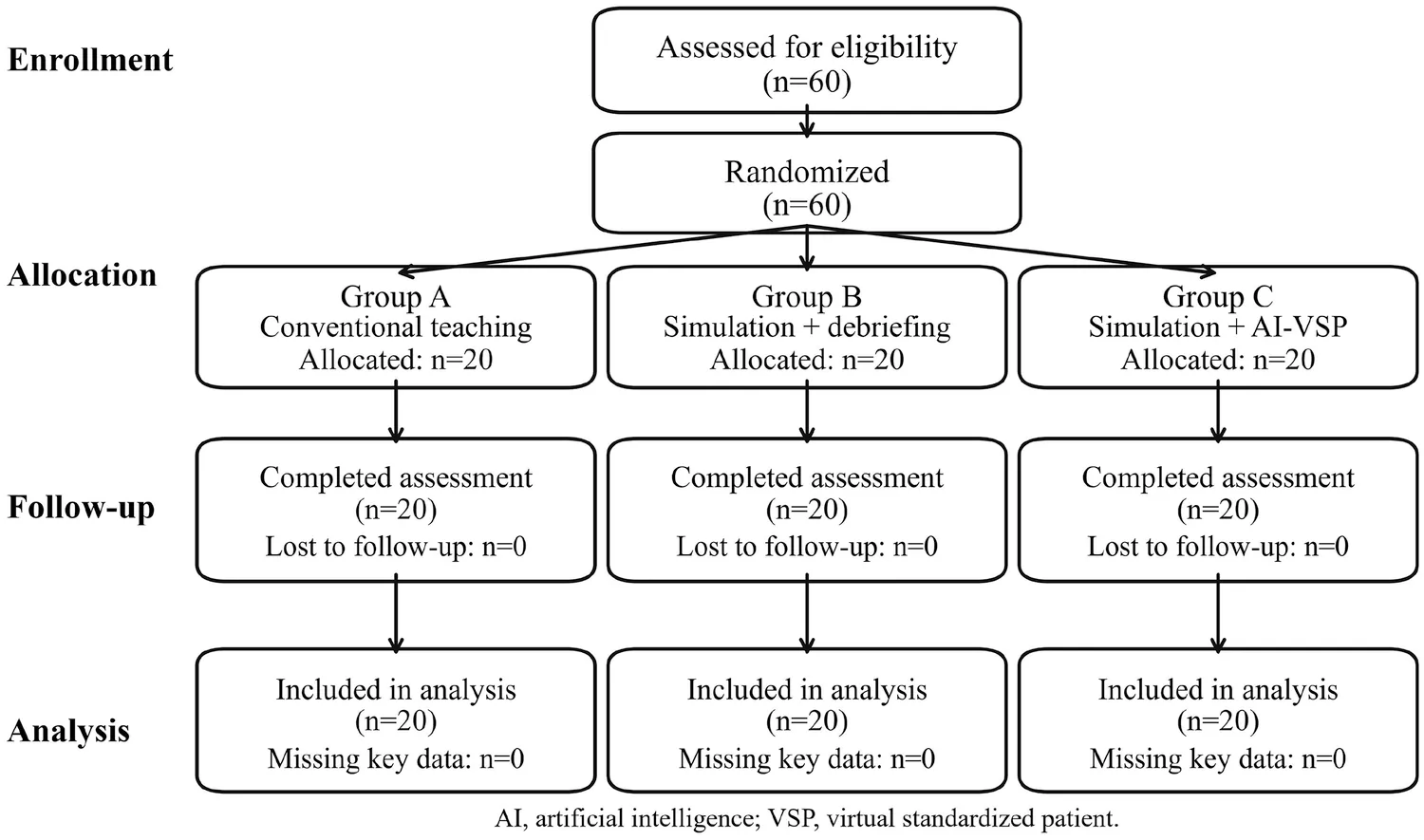
CONSORT-style participant flow diagram. The diagram summarizes enrollment, allocation, follow-up and analysis for the three randomized teaching groups. AI, artificial intelligence; VSP, virtual standardized patient.

Sixty eligible students were screened, enrolled, randomized and included in the final analysis. No participant withdrew and no key assessment data were missing (Figure 2). Baseline characteristics are summarized in Table 1. Mean age, sex distribution, pre-rotation theoretical scores and previous exposure to simulation training were similar across groups; no statistically significant between-group differences were observed for the reported baseline variables (all *P* ≥ 0.283).

**Table 1:** Baseline characteristics of the three groups.

Characteristic	Group A (*n* = 20)	Group B (*n* = 20)	Group C (*n* = 20)	Statistic	*P* value
Age, years	24.8 ± 0.9	24.8 ± 1.2	24.7 ± 0.9	F = 0.044	0.957
Sex, male/female	8/12	10/10	8/12	*χ*^2^ = 0.543	0.762
Pre-rotation theoretical score	79.2 ± 4.1	79.0 ± 3.2	77.4 ± 4.5	F = 1.291	0.283
Previous simulation training, yes/no	4/16	6/14	4/16	χ^2^ = 0.745	0.689

Values are mean ± standard deviation or counts. Group A, conventional teaching; group B, scenario-based simulation with structured debriefing; group C, scenario-based simulation combined with AI-driven VSP training. Continuous-variable statistics were calculated from participant-level data; categorical tests used the displayed counts.

### Intervention model

### Clinical performance outcomes

Baseline clinical performance did not differ significantly among groups (all *P* ≥ 0.157). Exploratory participant-level post-intervention comparisons favored group C for total simulated clinical performance, clinical decision-making and communication. After Bonferroni adjustment, group C outperformed group B in emergency management, clinical decision-making, communication and total performance. The group C vs. group B teamwork difference was not significant after adjustment. Professionalism did not differ among groups (Tables 2, 3).

**Table 2:** Clinical performance scores before and after intervention.

Domain	Time	Group A (*n* = 20)	Group B (*n* = 20)	Group C (*n* = 20)
Emergency management	Pre	12.2 ± 1.8	11.7 ± 1.7	11.9 ± 1.2
	Post	15.3 ± 2.4	16.2 ± 1.7	17.8 ± 1.6
Teamwork	Pre	12.5 ± 1.9	12.0 ± 1.6	12.0 ± 1.2
	Post	15.4 ± 1.9	16.1 ± 1.8	17.0 ± 1.5
Clinical decision making	Pre	12.7 ± 1.6	11.9 ± 1.2	12.8 ± 1.6
	Post	15.4 ± 1.8	15.7 ± 1.4	18.6 ± 1.1
Communication	Pre	13.6 ± 1.6	13.2 ± 1.5	13.4 ± 1.3
	Post	15.2 ± 1.5	15.9 ± 1.9	17.9 ± 1.3
Professionalism	Pre	12.9 ± 1.9	12.4 ± 1.5	12.2 ± 1.2
	Post	16.7 ± 1.8	16.6 ± 1.7	16.9 ± 1.5
Total simulated clinical performance	Pre	64.0 ± 7.6	61.2 ± 6.0	62.3 ± 4.9
	Post	78.0 ± 7.7	80.4 ± 6.5	88.2 ± 4.8

Values are mean ± standard deviation. The total simulated clinical performance score was the sum of the five 20-point domains.

**Table 3:** Participant-level post-intervention between-group comparisons.

Outcome	Post-intervention ANOVA	*P* value	Partial eta squared	C vs. A adjusted *P*	C vs. B adjusted *P*
Emergency management	F(2,57) = 8.55	<0.001	0.231	0.001	0.012
Teamwork	F(2,57) = 4.02	0.023	0.124	0.018	0.288
Clinical decision making	F(2,57) = 30.04	<0.001	0.513	<0.001	<0.001
Communication	F(2,57) = 16.09	<0.001	0.361	<0.001	<0.001
Professionalism	F(2,57) = 0.17	0.840	0.006	1.000	1.000
Total simulated clinical performance	F(2,57) = 13.72	<0.001	0.325	<0.001	<0.001
Theoretical examination	F(2,57) = 22.33	<0.001	0.439	<0.001	<0.001
Skills examination	F(2,57) = 40.13	<0.001	0.585	<0.001	<0.001
Teaching satisfaction	F(2,57) = 66.01	<0.001	0.698	<0.001	<0.001

F statistics, *P* values, partial eta squared and Bonferroni-adjusted *post hoc P* values were calculated from participant-level scores. These estimates remain exploratory because the baseline-adjusted ANCOVA was *post hoc* and participant-level repeated-measures models were not performed.

### Professional examination performance and teaching satisfaction

At baseline, theoretical and skills examination scores did not differ significantly among groups. After the intervention, group C had the highest theoretical and skills examination scores. Teaching satisfaction was also highest in group C; however, because the questionnaire was self-developed and potentially influenced by novelty, this result is interpreted as acceptability rather than proof of educational effectiveness (Table 4).

**Table 4:** Examination performance and teaching satisfaction.

Outcome	Time	Group A (*n* = 20)	Group B (*n* = 20)	Group C (*n* = 20)
Theoretical examination	Pre	79.2 ± 4.1	79.0 ± 3.2	77.4 ± 4.5
	Post	83.7 ± 4.2	86.9 ± 2.8	92.1 ± 4.8
Skills examination	Pre	75.9 ± 3.0	73.9 ± 4.9	74.6 ± 3.8
	Post	81.5 ± 2.9	85.8 ± 5.6	93.7 ± 4.3
Teaching satisfaction	Post only	81.1 ± 4.3	88.7 ± 3.6	94.2 ± 2.7

Values are mean ± standard deviation. Satisfaction was measured after the intervention only.

### Exploratory effect-size estimates

**Figure 3:**
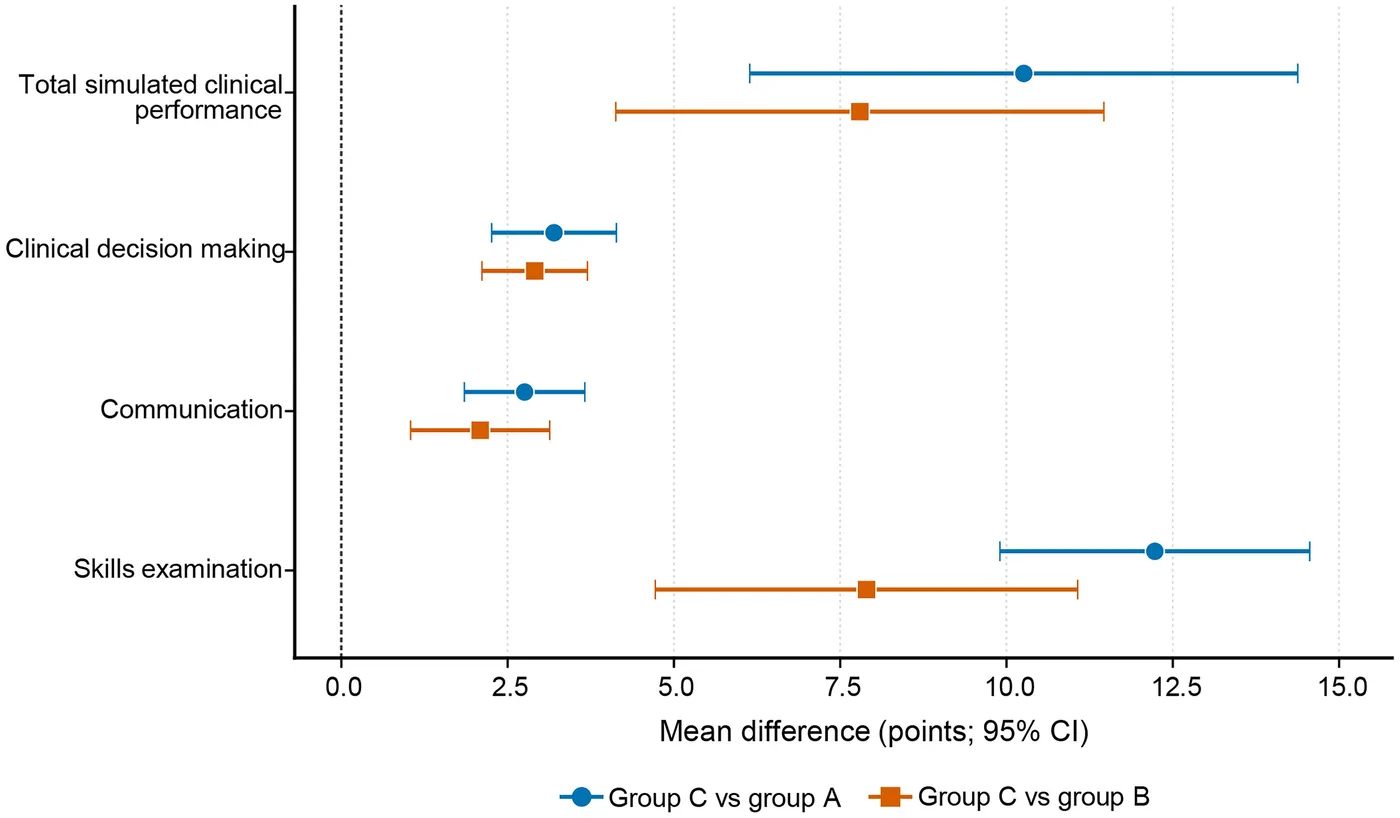
Exploratory mean differences in key post-intervention outcomes. Points show mean differences and whiskers show pooled-variance 95% confidence intervals calculated from participant-level data (*n* = 20 per group). Positive values favor the AI-driven VSP combined simulation model. Estimates should be interpreted as preliminary; the exploratory baseline-adjusted analysis is shown in Figure 6.

Exploratory participant-level estimates of post-intervention mean differences and standardized effect sizes favored group C for the key outcomes shown in Table 5 and Figure 3. Because this single-center pilot study included a modest sample, these estimates should be interpreted as hypothesis-generating and may overestimate the true effect.

**Table 5:** Exploratory post-intervention effect-size estimates for key outcomes.

Outcome	C vs. A mean difference (95% CI)	Cohen's *d*	C vs. B mean difference (95% CI)	Cohen's *d*
Total simulated clinical performance	10.3 (6.1 to 14.4)	1.59	7.8 (4.1 to 11.5)	1.36
Clinical decision making	3.2 (2.3 to 4.1)	2.18	2.9 (2.1 to 3.7)	2.35
Communication	2.8 (1.8 to 3.7)	1.95	2.1 (1.0 to 3.1)	1.28
Skills examination	12.2 (9.9 to 14.6)	3.36	7.9 (4.7 to 11.1)	1.59

Mean differences, 95% confidence intervals and Cohen's *d* values were calculated from participant-level data using pooled within-group variance for each independent-group comparison, with *n* = 20 per group. Positive values favor group C.

### Implementation fidelity and resource use

**Figure 4:**
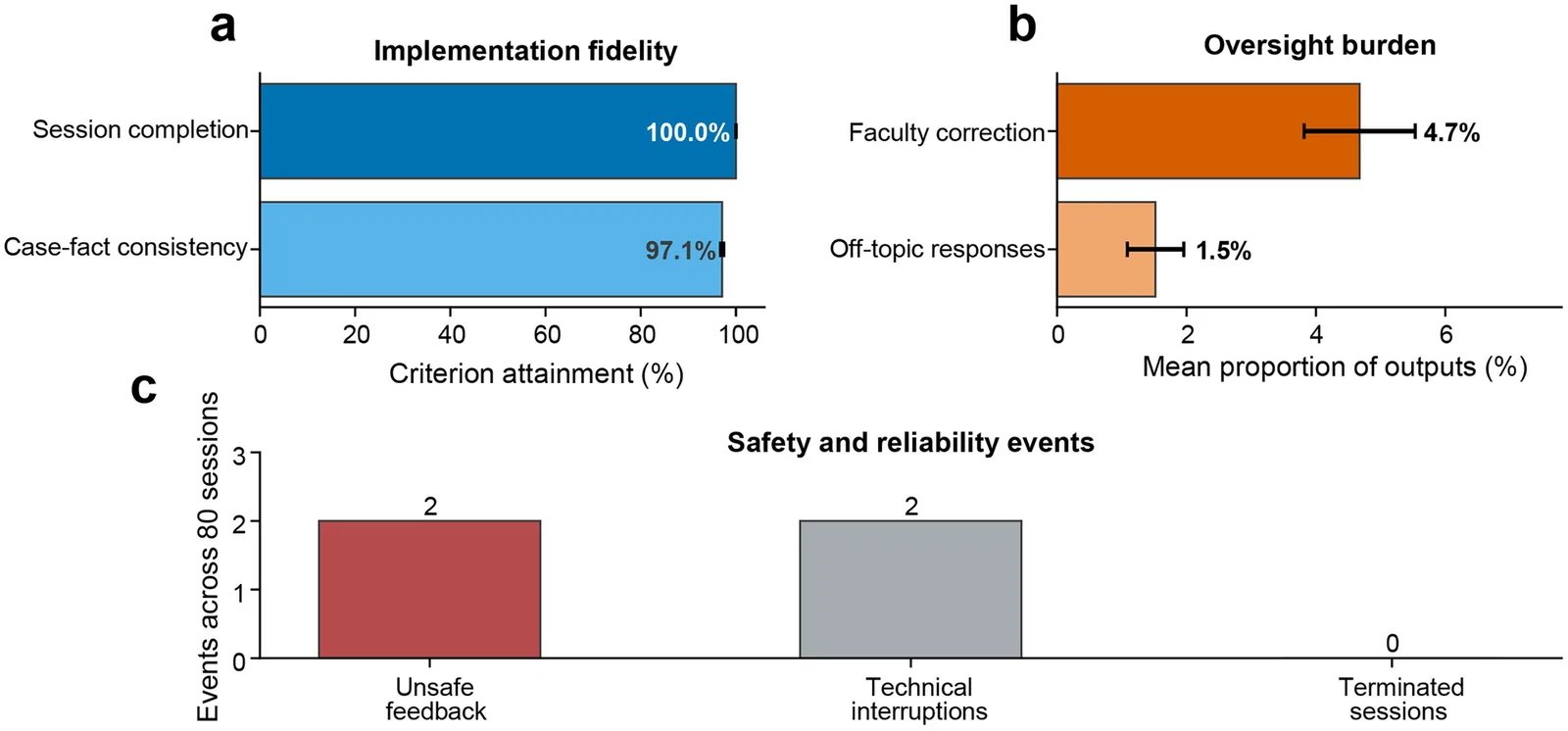
AI-VSP implementation fidelity, oversight and safety metrics. **(a)** Session completion and participant-level case-fact consistency. **(b)** Participant-level faculty-correction and off-topic-response rates. Bars show means and error bars show standard deviations (*n* = 20 participants); completion was 100% with no variation. **(c)** Total recorded event counts across 80 scheduled sessions.

**Figure 5:**
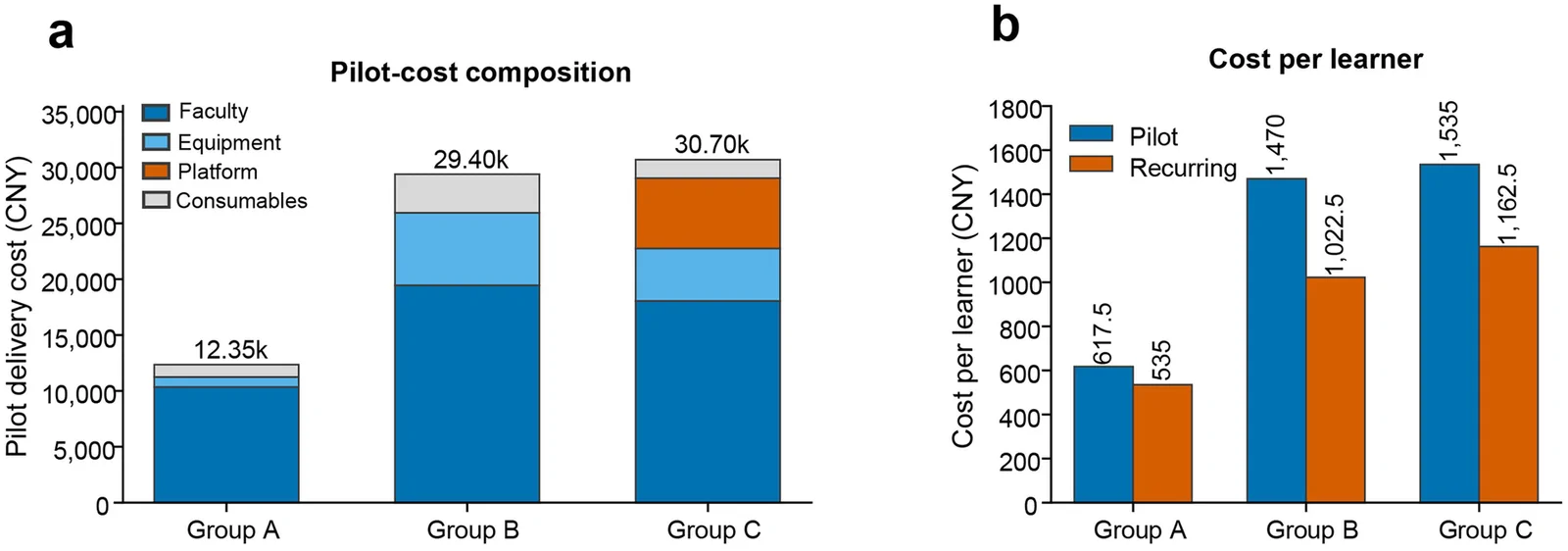
Educational delivery costs. **(a)** Stacked pilot-cost components for groups A–C. **(b)** Pilot and recurring cost per learner (*n* = 20 per group). Costs are expressed in CNY and describe implementation burden rather than cost-effectiveness.

Implementation-fidelity and resource-use data presented in Figures 4, 5 were derived from study records, attendance logs, internal teaching-system summaries, and cost records and are reported descriptively.

All 80 scheduled AI-VSP training sessions were completed. At the participant level (*n* = 20), mean case-fact consistency was 97.1% ± 0.3%, while mean faculty-correction and off-topic response rates were 4.7% ± 0.9% and 1.5% ± 0.4%, respectively. Two unsafe-feedback events and two technical interruptions totaling 10 min were recorded, and no session was terminated because of technical failure (Figure 4).

The pilot implementation costs for groups A, B, and C were CNY 12,350, CNY 29,400, and CNY 30,700, respectively. The corresponding pilot costs per learner were CNY 617.5, CNY 1,470.0, and CNY 1,535.0, while recurring implementation costs per learner were CNY 535.0, CNY 1,022.5, and CNY 1,162.5, respectively (Figure 5). These estimates describe implementation burden and should not be interpreted as a formal cost-effectiveness analysis.

### Exploratory baseline-adjusted analyses

**Figure 6:**
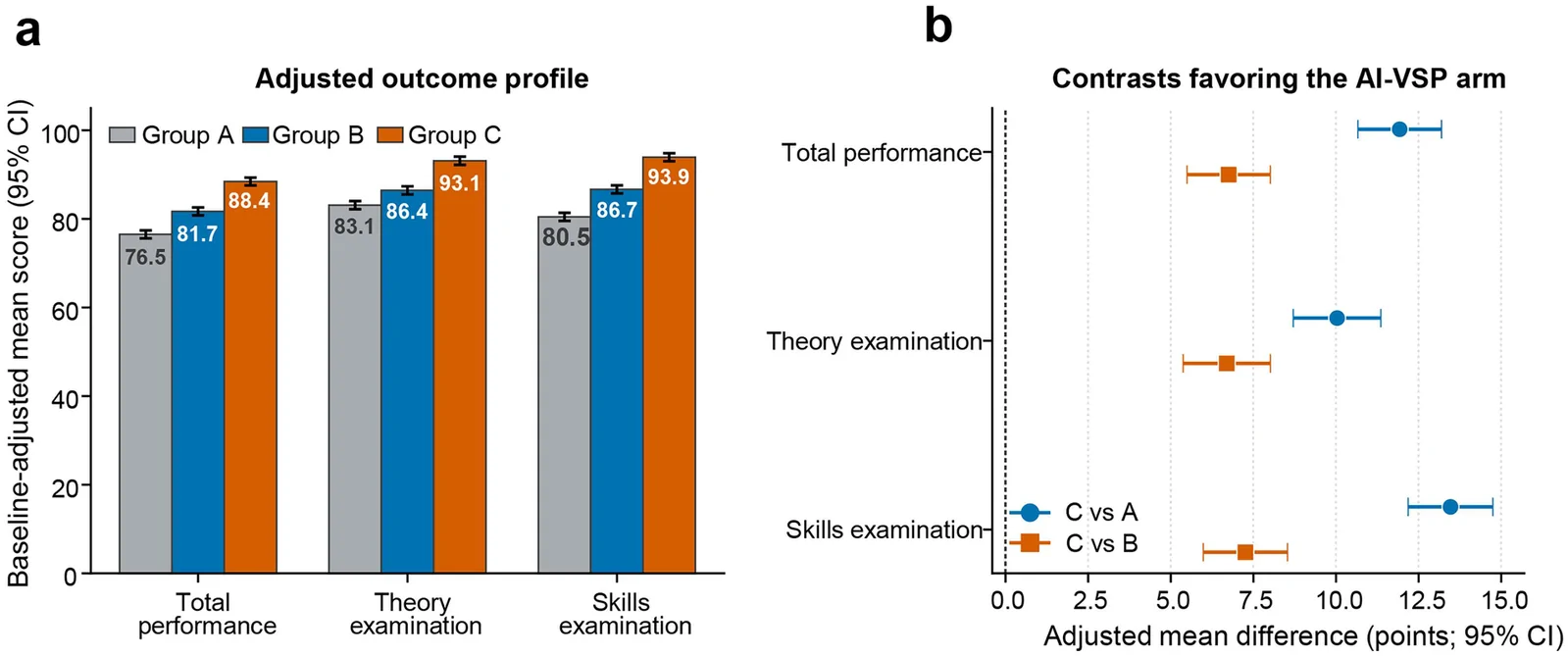
Exploratory baseline-adjusted outcomes. **(a)** ANCOVA-adjusted means and 95% confidence intervals for total performance, theory and skills. **(b)** Adjusted group C versus group A and group C versus group B differences with 95% confidence intervals. Models included group and the corresponding baseline score (*n* = 60; 20 per group; residual df = 56). Group-by-baseline interaction tests supported the homogeneous-slopes assumption (all *P* ≥ 0.207). The analysis was *post hoc* and exploratory.

In exploratory *post hoc* ANCOVA, the ordering of the three groups remained consistent across all three outcomes, with group A < group B < group C. Adjusted means for total clinical performance were 76.5, 81.7, and 88.4; for theoretical examination, 83.1, 86.4, and 93.1; and for skills examination, 80.5, 86.7, and 93.9 for groups A, B, and C, respectively. Group effects were significant for total clinical performance [F(2,56) = 179.84], theoretical examination [F(2,56) = 118.76], and skills examination [F(2,56) = 222.15; all *P* < 0.001]. For all three outcomes, the 95% confidence intervals for the comparisons of group C with groups A and B did not cross zero (Figure 6). These analyses were exploratory and were not prespecified.

## Discussion

This randomized pilot study compared three time-matched teaching pathways. The group C pathway, in which four 1-hour AI-VSP sessions replaced 4 h of conventional case discussion while scenario-based simulation and structured debriefing were retained, was associated with better short-term performance on aligned anesthesiology crisis tasks than either comparator. The largest between-group differences involved total simulated clinical performance, clinical decision-making, communication, theoretical examination and skills examination. The pattern is educationally plausible but preliminary because the study was single-center, modest in size, short-term and limited to simulated-performance outcomes.

We intentionally avoid interpreting these findings as definitive evidence of broad clinical competency. Although the assessment case was parallel rather than identical to the training cases, it remained within the same perioperative bradycardia and hypotension crisis domain and preserved the same critical action categories. This degree of alignment is appropriate for early educational evaluation, but it may also mean that gains reflect short-term scenario-specific practice, improved familiarity with crisis logic or performance optimization rather than durable transfer to workplace practice. Future studies should use multiple independently developed parallel assessment cases, delayed retention testing and workplace-based outcomes.

The decision-making findings are consistent with theories of deliberate practice and mastery learning. Within the integrated teaching pathway, AI-VSP sessions allowed learners to repeatedly gather information, interpret evolving physiological data, formulate differential diagnoses, prioritize actions and receive structured feedback. These activities may have reinforced CRM-relevant cognitive processes while complementing the multi-role crisis-management and coordination practice delivered through scenario-based simulation and structured debriefing (13–16, 36). This interpretation is also supported by systematic reviews in health professions education showing that virtual patients can improve knowledge, clinical reasoning and skills compared with no intervention (19, 20). More recent empirical studies have reported improved history-taking and medical-interview performance after training with LLM-based digital patients or AI-simulated patients (22, 37). The present findings add evidence from perioperative crisis education, where communication must be integrated with evolving physiological information and time-critical clinical decisions.

The communication findings are also consistent with the emerging AI-VSP literature. Conventional standardized patients provide responsive dialogue and interpersonal realism but, without additional simulation technology, have limited capacity to reproduce monitor changes or treatment-linked physiological deterioration. Scenario-based simulation can reproduce physiological deterioration, monitor changes and clinical workflow, but it often provides limited patient dialogue (12). AI-driven VSP interaction may provide more frequent practice in patient-centered questioning, concise explanation and response to patient concerns while linking dialogue to scripted vital-sign trajectories and rule-based responses to clinical actions. This combination may be especially relevant to preoperative assessment and perioperative crisis management, where incomplete histories or unclear communication can delay risk recognition and treatment. The communication outcome should therefore be interpreted primarily as patient-facing and information-synthesis practice rather than as a complete measure of multiprofessional CRM communication.

Viewed within a CRM framework, the domain-specific findings provide a more precise interpretation of the combined intervention. This interpretation is consistent with anesthesia CRM studies showing that realistic crisis practice and constructive debriefing support the development of non-technical performance (3, 4, 10). The clearest additional advantages of group C involved clinical decision-making and communication, both of which were directly practiced during AI-VSP sessions. In contrast, the group C vs. group B difference in teamwork was not significant after adjustment for multiple comparisons, and professionalism did not differ among groups. This pattern suggests a selective and complementary educational contribution: embedded AI-VSP practice may strengthen selected cognitive and communication processes, whereas leadership, role allocation, shared workload management and coordination across clinical roles are more fully addressed through scenario-based simulation and structured debriefing.

The automated performance reports may also have value for educators. By summarizing key questions, decision timing, omitted critical actions and triggered error pathways, the reports provide a case-aligned structure for the AI-VSP-specific feedback review. Previous work found high overall agreement between automated and human feedback while also identifying category-specific discrepancies, reinforcing the value of faculty verification (38). In the present study, faculty used the reports to focus discussion on performance relative to the case objectives; this review was additional to, and distinct from, the GAS debriefing following scenario-based simulation. By reducing the need to reconstruct the session from memory, the reports may support more consistent feedback and allow faculty to focus on targeted coaching. When retained in an auditable format, these data may also support longitudinal learning analytics. The present pilot focused on learner outcomes; future implementation studies should evaluate faculty time, feedback consistency, debriefing quality and the educational usefulness of report-based analytics.

The AI component requires careful implementation. AI-generated educational interactions can be variable, opaque and vulnerable to hallucination, automation bias or inappropriate learner over-trust (23, 24, 26–28). AI-VSP systems should therefore be deployed as faculty-supervised simulation tools with scenario constraints, educator-approved content, privacy safeguards, documented failure-handling procedures and explicit learner instruction that AI outputs are for training only. Departments considering implementation should pilot the system with faculty review, monitor technical failures, log learner exposure and update case scripts according to curriculum needs.

Teaching satisfaction was higher in the AI-VSP group, supporting acceptability but not effectiveness. Satisfaction is subjective and may be influenced by novelty, perceived innovation or learner expectations. The satisfaction questionnaire was locally developed, and internal consistency was not calculated. Future evaluations should combine satisfaction with objective performance, qualitative learner feedback, cost and workload data, and implementation outcomes.

### Strengths and limitations

The study has several strengths: a randomized three-arm pilot design, equalized teaching exposure, video-based performance assessment, blinded independent scoring, and inclusion of both simulation-alone and conventional teaching comparators. The intervention also addresses a practical educational challenge in anesthesiology: the need for repeated practice in low-frequency, high-risk crisis situations.

Several limitations are important. First, the study was conducted at a single center with 60 participants, and the effect-size estimates are unstable and likely to be inflated. Second, although total instructional time was equalized and the AI-VSP sessions replaced rather than added to conventional case discussion, the trial compared bundled teaching pathways. The group C vs. group B contrast therefore represents the effect associated with substituting the complete AI-VSP instructional component, including repeated interactive practice, automated formative reports and faculty review, for conventional case discussion, rather than the isolated effect of the AI technology itself. The group C vs. group A contrast reflects the combined teaching pathway and is not AI-specific. Third, only short-term outcomes were measured; no retention or workplace performance endpoints were available. Fourth, although the assessment case was parallel and not identical to the training cases, it remained aligned with the same crisis domain. The results therefore represent aligned simulated performance rather than broad clinical transfer. Fifth, the primary outcome used a locally developed rubric. Although content validity, rater calibration and inter-rater reliability were promising, selection of an exact score within a behavioural band remained a global rater judgement and may retain residual subjectivity; external validation remains necessary. Sixth, the participant-level ANCOVA was *post hoc* and not prespecified, and mixed-effects sensitivity analyses were not performed. Seventh, complete raw AI interaction logs were unavailable for independent dialogue-level reanalysis. Eighth, the AI-VSP platform is reported at the functional and governance level because a public model-version identifier was unavailable, which may limit replication. Finally, educators and learners could not be blinded, and novelty effects may have influenced engagement and satisfaction. In addition, although the competency rubric was informed by ANTS, it was not the formal ANTS instrument or a validated comprehensive CRM measure, and no separate team-level CRM score was collected.

### Implications for practice and research

For departments with limited access to repeated high-fidelity simulation, AI-driven VSP training may be embedded within a combined scenario-based simulation and structured-debriefing pathway as a complementary format for repeated CRM-relevant cognitive and communication practice. A safe implementation model should include educator-approved scripts, faculty monitoring, data minimization, standardized debriefing and routine technical review. Future trials should recruit larger multicenter samples, preregister analytic plans, retain auditable participant-level data for prespecified baseline-adjusted modeling, fully archive AI interaction logs, report system details using CONSORT-AI-informed elements, use independent parallel assessment cases, include delayed follow-up, and evaluate resource use and workplace performance. Future studies should also prespecify CRM-related outcomes and incorporate externally validated non-technical skills instruments and team-level assessments. Future implementation studies should also quantify faculty time, feedback consistency and the validity and educational usefulness of report-based learning analytics.

## Conclusions

Scenario-based simulation combined with AI-driven VSP training was associated with better short-term performance on aligned anesthesiology crisis tasks in this randomized pilot. The findings support feasibility and preliminary educational promise, particularly for clinical decision-making and communication. These findings concern selected CRM-relevant domains within aligned simulated crisis performance and do not establish comprehensive CRM competence, broad clinical competence or durable transfer to clinical practice. Future multicenter trials should use transparent AI reporting, prespecified participant-level analyses, independent parallel assessment cases, delayed retention testing and workplace outcomes.

## Data Availability

The original contributions presented in the study are included in the article/Supplementary Material, further inquiries can be directed to the corresponding author/s.
