# Supplementary material for: AI-driven virtual standardized patients combined with scenario-based simulation in anesthesiology training: a randomized pilot study
Source: Front Med (Lausanne). 2026 Sep 8;13:1927674. doi: 10.3389/fmed.2026.1927674 (PMC13593489; doi:10.3389/fmed.2026.1927674)
Supplement: Supplementary file 1 [file Supplementaryfile1.docx]

**AI-driven virtual standardized patients combined with scenario-based simulation in anesthesiology training: a randomized pilot study**

Original Article

Xiaohua Wang^1#^, Aming Sang^1#^, Jing Zhang^1#^, Hexiao Tang^2^, Yujia Liu^4^, Zirui Zhao^4^, Jinping Liu^3*^, Ming Xu^2*^, Xinyi Li^1*^

1 Department of Anesthesiology, Zhongnan Hospital of Wuhan University, Wuhan 430071, China

2 Department of Thoracic Surgery, Zhongnan Hospital of Wuhan University, Wuhan 430071, China

3 Department of Cardiovascular Surgery, Zhongnan Hospital of Wuhan University, Wuhan 430071, China

4 School of Basic Medical Sciences, Wuhan University, Wuhan 430071, Hubei Province, China

^#^Co-first authors: Xiaohua Wang, Aming Sang, and Jing Zhang contributed equally to this work.

*Corresponding authors:

Jinping Liu, Department of Cardiovascular Surgery, Zhongnan Hospital of Wuhan University, Wuhan 430071, Hubei Province, China. Email: 692005504@qq.com.

Ming Xu, Department of Thoracic Surgery, Zhongnan Hospital of Wuhan University, Wuhan 430071, Hubei Province, China. Email: xuming@znhospital.cn.

Xinyi Li, Department of Anesthesiology, Zhongnan Hospital of Wuhan University, Wuhan 430071, Hubei Province, China. Email: lxy08272021@126.com.

# **Supplementary Material**

*Supplementary Table S1. Clinical performance assessment rubric.*

| **Domain** | **Score band** | **Behavioral anchor** |
| --- | --- | --- |
| Emergency management | 0-5 | Fails to recognize crisis or provides unsafe/absent response; does not initiate airway, breathing, circulation assessment. |
|  | 6-10 | Recognizes abnormal vital signs but response is delayed, incomplete, or poorly prioritized. |
|  | 11-15 | Generally appropriate recognition and initial treatment; some omissions in escalation, monitoring, or reassessment. |
|  | 16-20 | Rapid recognition, prioritized treatment, timely escalation, continuous reassessment, and safe adaptation to changing data. |
| Teamwork | 0-5 | No role allocation, unclear commands, poor awareness of team resources. |
|  | 6-10 | Partial role allocation but inconsistent closed-loop communication and coordination. |
|  | 11-15 | Adequate team coordination with minor omissions in closed-loop communication or workload distribution. |
|  | 16-20 | Clear leadership, role allocation, closed-loop communication, and effective collaboration with nurses/surgeons. |
| Clinical decision making | 0-5 | Inaccurate diagnosis, unsafe priorities, or failure to integrate available information. |
|  | 6-10 | Recognizes some information but differential diagnosis and management plan are incomplete. |
|  | 11-15 | Reasonable differential diagnosis and treatment priorities with some delay or missed alternatives. |
|  | 16-20 | Accurate information integration, prioritized differential diagnosis, timely treatment, and dynamic adjustment. |
| Communication | 0-5 | Incomplete history taking, unclear explanation, or poor response to patient/team concerns. |
|  | 6-10 | Obtains limited information and communicates inconsistently or ambiguously. |
|  | 11-15 | Generally clear patient/team communication with minor omissions. |
|  | 16-20 | Structured history taking, concise explanation, patient-centered response, and clear team communication. |
| Professionalism | 0-5 | Unsafe attitude, poor responsibility, or disregard for patient safety and ethics. |
|  | 6-10 | Basic professionalism but inconsistent safety awareness or accountability. |
|  | 11-15 | Appropriate responsibility and patient-safety awareness with minor deficiencies. |
|  | 16-20 | Consistent professionalism, ethical awareness, responsibility, and reflective practice. |

Each domain was scored from 0 to 20 points. The total score ranged from 0 to 100 points. Raters were instructed to assign scores according to the behavioral anchors and overall observed performance.

*Supplementary Table S2. Standardized simulation and assessment scenario checklist.*

| **Component** | **Description** |
| --- | --- |
| Training scenario theme | Perioperative bradycardia and hypotension crisis during anesthesia-related training. |
| Learning objectives | Recognize hemodynamic instability; obtain relevant perioperative information; identify likely causes; initiate prioritized management; communicate with team members; reassess response. |
| Initial state | Stable perioperative patient before crisis trigger; baseline history and vital signs provided through scenario materials and/or virtual patient interaction. |
| Trigger event | Progressive bradycardia and hypotension displayed by monitor changes, patient responses, and scenario cues. |
| Expected critical actions | Confirm monitor accuracy; assess airway/breathing/circulation; call for help; inform surgical/nursing team; consider anesthetic depth, vagal reflex, hypovolemia, drug effect, bleeding, or other causes; initiate appropriate medication and fluid/vasopressor management; reassess. |
| Debriefing focus | Crisis recognition, differential diagnosis, decision priorities, communication clarity, closed-loop teamwork, omissions, and action plan for improvement. |
| Assessment relationship to training | Parallel but distinct assessment case. The assessment preserved the same critical action domains (crisis recognition, airway-breathing-circulation assessment, differential diagnosis, treatment prioritization, team communication, and reassessment) but used a different fictional patient profile, comorbidity/distractor information, trigger timing, and cue sequence from the training cases. |

*Supplementary Table S3. Teaching satisfaction questionnaire structure.*

| **Dimension** | **Score range** | **Example content assessed** |
| --- | --- | --- |
| Teaching content arrangement | 0-20 | Relevance, clarity, and appropriateness of teaching content. |
| Teaching method | 0-20 | Suitability of teaching format and alignment with learning objectives. |
| Interaction and engagement | 0-20 | Learner participation, interactivity, and immersion. |
| Perceived learning gain | 0-20 | Self-perceived improvement in knowledge, skills, and clinical reasoning. |
| Overall satisfaction | 0-20 | Overall acceptability and perceived value of the teaching model. |

Total score ranged from 0 to 100 points. The questionnaire was locally developed and internal consistency was not calculated; therefore, it was interpreted only as an acceptability measure.

*Supplementary Table S4. AI-VSP technical specification and governance reporting.*

| **Reporting item** | **Reported information** |
| --- | --- |
| Platform identity and developer | CliniTrek, a generative AI-driven clinician-patient intelligent interaction and competency assessment system developed by Wuhan Kelin Siyuan Information Technology Co., Ltd. (Wuhan, China). |
| Deployment and study configuration | The platform was locally deployed on password-protected institutional teaching computers at Zhongnan Hospital of Wuhan University and configured with faculty-authored anesthesiology cases. The study configuration was locked from January to June 2026. A public model-version identifier was not available from the developer; the dialogue engine, case content, trigger rules, vital-sign trajectories, and feedback templates remained fixed during the intervention period. |
| Interaction modalities | Mandarin Chinese voice interaction using speech-to-text and text-to-speech functions, an on-screen dialogue transcript, displayed vital signs and monitor alarms, spoken management instructions, and an automated post-session report. |
| Session and agent configuration | Each session was completed by one learner. Depending on the case script, the software-mediated roles included a virtual patient, a virtual nurse, and, where relevant, a family agent. The virtual nurse acknowledged management instructions and activated predefined actions and corresponding physiological responses. No learner teammates or human actors participated as in-scenario team members. Faculty supervised platform use but did not perform a scenario role. |
| Scenario engine and branching | The platform combined a scenario-constrained large-language-model dialogue engine with a faculty-authored case fact bank, scripted vital-sign trajectories, and rule-based trigger events. Learner questions and management instructions activated predefined information-release or scenario branches. Critical deterioration, treatment responses, and stopping criteria were governed by scripted rules rather than unrestricted model generation. |
| Feedback generation and report content | Recorded learner actions were mapped to faculty-authored critical-action checklist items and predefined timing and sequence rules. The report summarized decision timing, omitted critical actions, delayed or potentially unsafe actions, activated error pathways, and template-based improvement points. Although CliniTrek includes competency assessment functions, its reports were used only for formative feedback and were excluded from all study outcome assessments. The platform did not assign ANTS or CRM scores. |
| Feedback delivery and relation to debriefing | The automated report was provided at the end of each AI-VSP session and reviewed by faculty with the learner within the scheduled session. Faculty corrected inaccurate, incomplete, unsafe, or off-topic comments when necessary. This AI-VSP-specific feedback was separate from and did not replace or alter the faculty-led GAS debriefing after high-fidelity simulation. |
| Representative feedback examples | Illustrative excerpts derived from faculty-approved feedback templates, rather than participant-level outcome data: (1) "Bradycardia recognized; treatment decision delayed; post-intervention reassessment omitted." (2) "Hypotension was identified, but the differential diagnosis was incomplete and closed-loop confirmation was omitted." |
| Human oversight and safety controls | Anesthesiology faculty approved the case scripts, learning objectives, critical actions, trigger rules, and feedback templates before implementation. AI responses were restricted to the scripted roles and predefined case information. Vital-sign changes and critical events were controlled by scenario rules. Faculty remained available during sessions and corrected unsafe or irrelevant output when required. |
| Privacy and data governance | Learners were instructed not to enter real patient identifiers. Training records were coded using study identifiers and stored on password-protected institutional computers or local teaching-system storage. Access was restricted to authorized teaching faculty and research personnel. |
| Implementation fidelity and burden | All 20 group C learners completed four 1-hour sessions, yielding 80 completed AI-VSP sessions. No session was terminated because of technical failure. Implementation required teaching computers, a microphone or headset, faculty-authored cases, scheduled teaching time, and faculty review of post-session reports. Complete raw transcripts and time-stamped interaction logs were not retained for all sessions. |

*Supplementary Table S5. Format, constructs, administration and scoring of the theoretical and skills examinations.*

| **Assessment** | **Format and scoring** | **Construct and interpretation** |
| --- | --- | --- |
| Theoretical examination | Faculty-developed written test comprising multiple-choice, short-answer and case-analysis items. Responses were scored using a prespecified answer key and marking rubric; item scores were summed to 100. | Factual and applied knowledge of the recognition and initial management of perioperative bradycardia and hypotension. CRM principles were not a separate domain, and behavioural CRM competence was not directly assessed. |
| Skills examination | Structured practical assessment scored with a standardized checklist; points for correct completion and sequencing of predefined steps were summed to 100. | Individual anesthesiology procedures, monitoring interpretation, initial emergency management and reassessment. It assessed technical and applied clinical performance and was distinct from the video-recorded primary outcome assessment. |

Both examinations were completed individually before and after the intervention under faculty supervision, using standardized instructions and scoring procedures; no coaching was provided during testing. CRM, Crisis Resource Management.
